# An Animal Explant Model for the Study of Human Cutaneous Squamous Cell Carcinoma

**DOI:** 10.1371/journal.pone.0076156

**Published:** 2013-10-08

**Authors:** Daniel A. Belkin, Jie Chen, Jonathan L. Mo, James S. Rosoff, Sagit Goldenberg, Dix P. Poppas, James G. Krueger, Miriam Herschman, Hiroshi Mitsui, Diane Felsen, John A. Carucci

**Affiliations:** 1 Department of Dermatology, Weill Cornell Medical College, New York, New York, United States of America; 2 Institute for Pediatric Urology, Department of Urology, Weill Cornell Medical College, New York, New York, United States of America; 3 Laboratory for Investigative Dermatology, Rockefeller University, New York, New York, United States of America; 4 Ronald O. Perelman Department of Dermatology, New York University Langone Medical Center, New York, New York, United States of America; Ohio State University Medical Center, United States of America

## Abstract

We established a human tissue explant model to facilitate study of cutaneous squamous cell carcinoma. We accomplished this by implanting debulked SCC, from surgical discard, into nude rats. Human SCC remained viable and continued to proliferate for at least 4 weeks and showed evidence of neovascularization. At 4 weeks, SCC implants showed a trend toward increased PCNA positive cells compared to fresh SCC cells/mm^2^ tissue) supporting continued proliferation throughout engraftment. Von Willebrand's Factor (VWF) positive cells were found within implants and likely represented rat vessel neovascularization. Human Langerhans' (Langerin+) cells, but no T cells (CD3+, CD8+, FoxP3+), macrophages (CD163), or NK cells (NKp46), were present in SCC implants at 4 weeks. These findings support the possibility that LCs fail to migrate from cutaneous SCC and thus contribute to lack of effective antitumor response. Our findings also provide a novel model system for further study of primary cutaneous SCC.

## Introduction

Animal model systems of cancer are necessary to understand the complex biology of tumors and to develop novel translational approaches to treatment. Animal tumor models come closer than *in vitro* study systems to reproducing the complexity of naturally-occurring *in situ* cancers [1]. An ideal animal model would allow replication of tumor-host interactions, e.g., immune response, angiogenesis, invasion, and metastasis, while being reproducible, easy to use, accessible to genetic and immunologic manipulations, and characterized by rapid progression [1]. No animal model can wholly fulfill these criteria, and a balance must be struck between fidelity to human conditions and practical considerations. Since many basic questions in cancer remain unresolved and many therapeutics fail during development, additional relevant animal model systems are needed [2].

Human xenograft models have become the gold standard for drug development in oncology [1]. Xenograft models involve either the inoculation of immune-deficient rodents with human cancer cell lines [3], [4], [5] or the surgical engraftment of whole human tissue [6]. Unlike cell line derived xenografts, explants of fresh patient material show architecture, cell morphology, and molecular characteristics similar to the native tumor [1]. In head and neck squamous cell carcinoma (HNSCC), patient-derived tumor explants grow as solid tumors with many histological characteristics of the parent tumor [7]. Furthermore, models of this sort have the benefit of including extracellular tissue elements. According to the contemporary view, tumor progression is a result of interactions between cancer cells and their stromal microenvironment [8], [9]. Infiltrating immune cells [10], [11], [12], [13], [14] as well as fibroblasts and extracellular matrix [15], [16] play vital roles in determining tumor behavior. Our group has shown stromal elements to be extensively active in cutaneous squamous cell carcinoma (SCC) [17], [18], [19]. This understanding underlies efforts to more accurately recapitulate the human tissue context of tumor behavior in animal models [2].

Current human tissue animal models for SCC include subcutaneous injection of SCC cell lines [20], [21] and engraftment of genetically engineered SCC-bearing skin [2]. No xenograft model using fresh patient-derived whole tumor exists for cutaneous SCC, which is the second most common human cancer [22].

In this paper, we establish a human tissue explant model for SCC using patient-derived whole tumor. This easily replicable model will serve as a model for evaluating novel treatment approaches and may ultimately allow for the use of custom-made patient-specific protocols to treat inoperable and metastatic SCC.

## Results

### Tumor tissue remains viable macroscopically four weeks after transplantation

**Figure 1:**
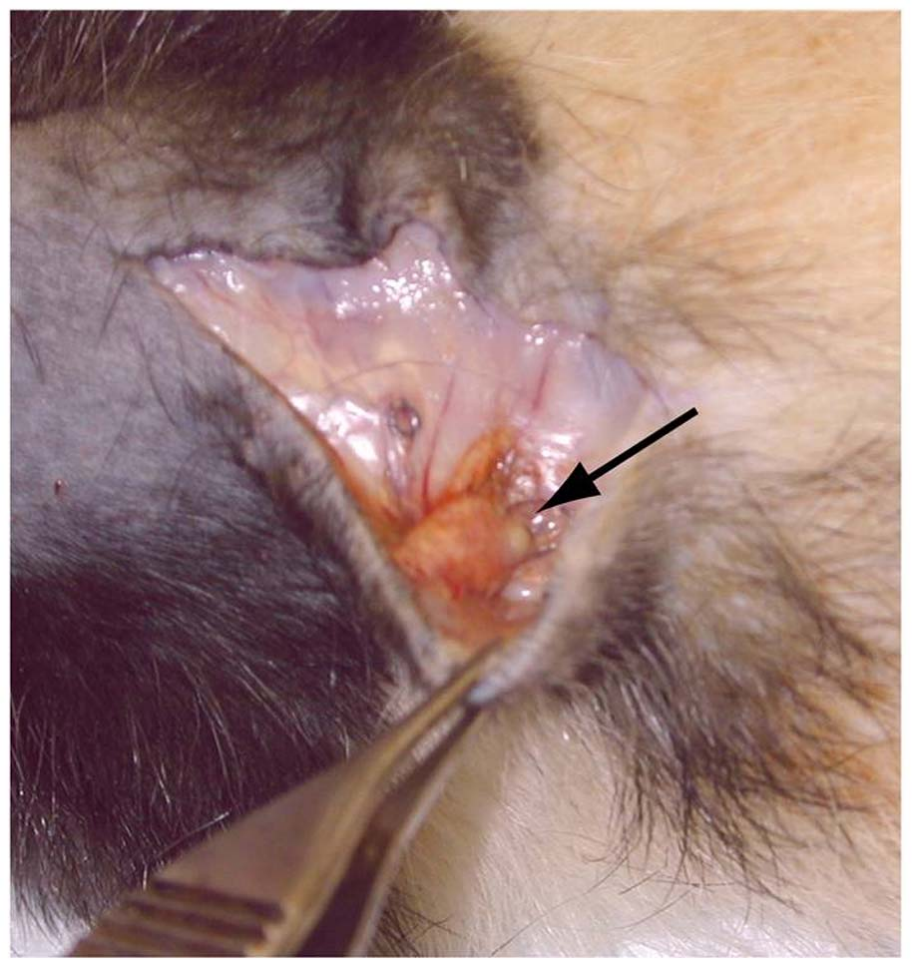
Tumor tissue remains viable macroscopically four - weeks after transplantation. The initial incision on the dorsum of the rat was reopened, the area dissected, and the grossly visible tumor removed en bloc, along with rat skin and fascia, for frozen and paraffin preservation. On average, tumors were about 40% smaller after four weeks, which may reflect initial contraction before vessel ingrowth presumably occurs.

A model was developed to grow human cutaneous SCC in nude rats [see Methods for details]. Briefly, rats were anesthetized and bulk human SCC, including epidermis and dermis, were implanted subcutaneously. Tissues remained in situ for 4 weeks and were then harvested. Tumors were successfully grafted in 8 of 9 rats. When the dorsal incisions were reopened four weeks after grafting, gross inspection revealed tumors that could be readily discriminated from the surrounding normal rat tissue. Mean tumor dimensions upon implantation were on average 84 mm^3^. After four weeks, mean volume from en bloc specimens was 36 mm^3^, which represents a contraction to ∼43% of the initial volume. Complications related to the grafting procedure were minor and involved several instances of small wound dehiscence that were promptly repaired. Figure 1 demonstrates a tumor graft seen grossly immediately prior to harvest.

### Human cutaneous squamous cell carcinoma was identified in grafts

**Figure 2:**
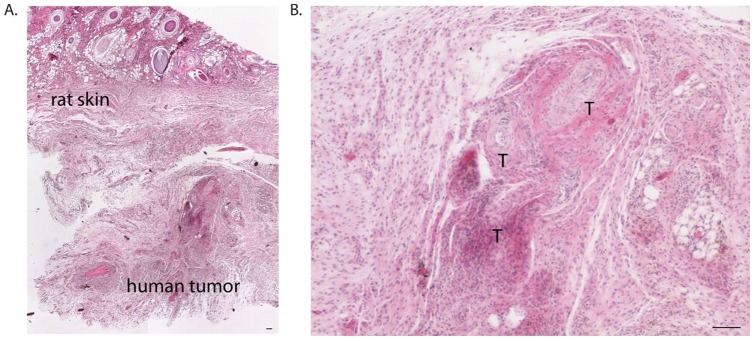
SCC retains its “signature pathologic” characteristics after implantation. Human tissue and rat tissue were readily distinguishable based on architecture (2A). Higher power reveals SCC-like tumor nests (T) (2B). Scale bars  = 100 µm.

**Figure 3:**
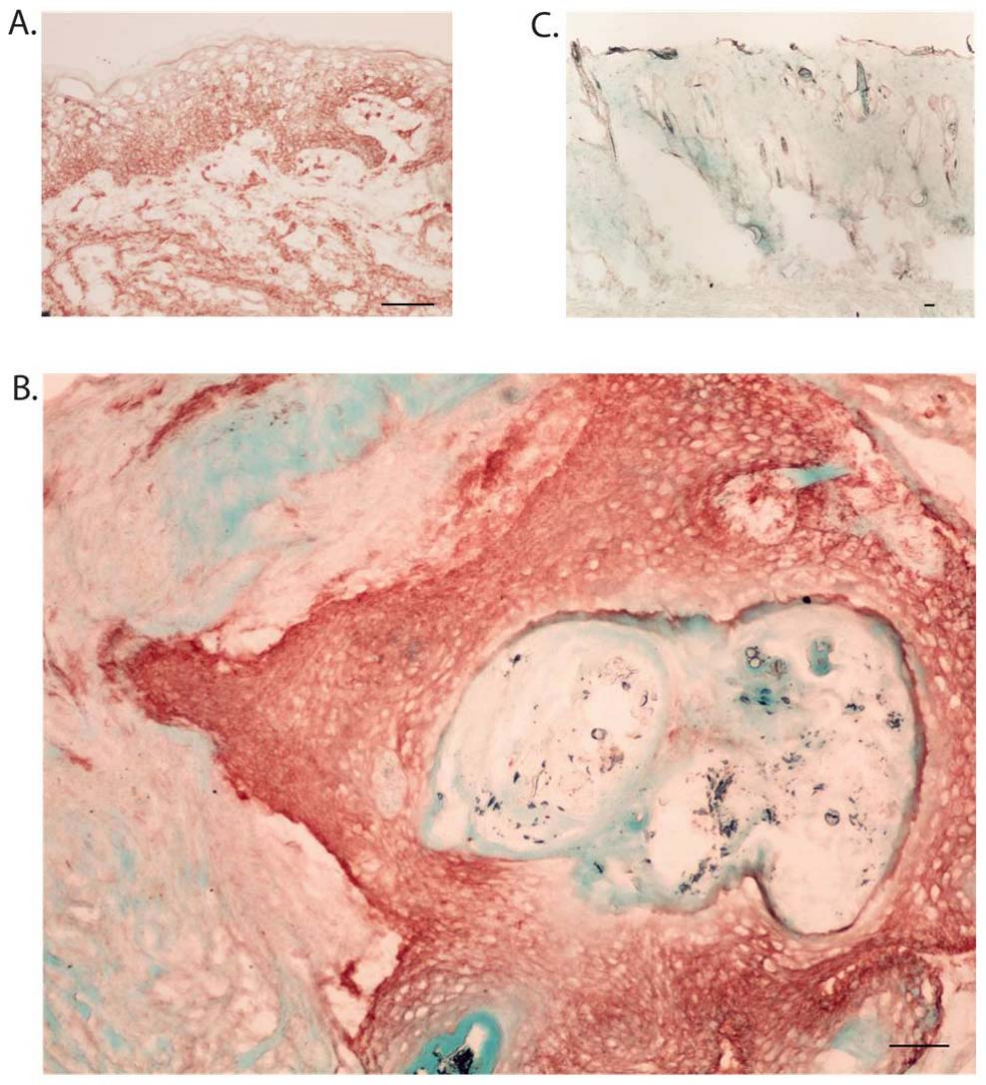
SCC retains its human markers four weeks after transplantation in the nude rat. We used HLA I as a marker for human tissue. Fresh non-transplanted human SCC stains with HLA I (3A), whereas rat tissue does not (3B). After four weeks, tumor nests stain for HLA I (3C). Scale bars  = 100 µm.

**Figure 4:**
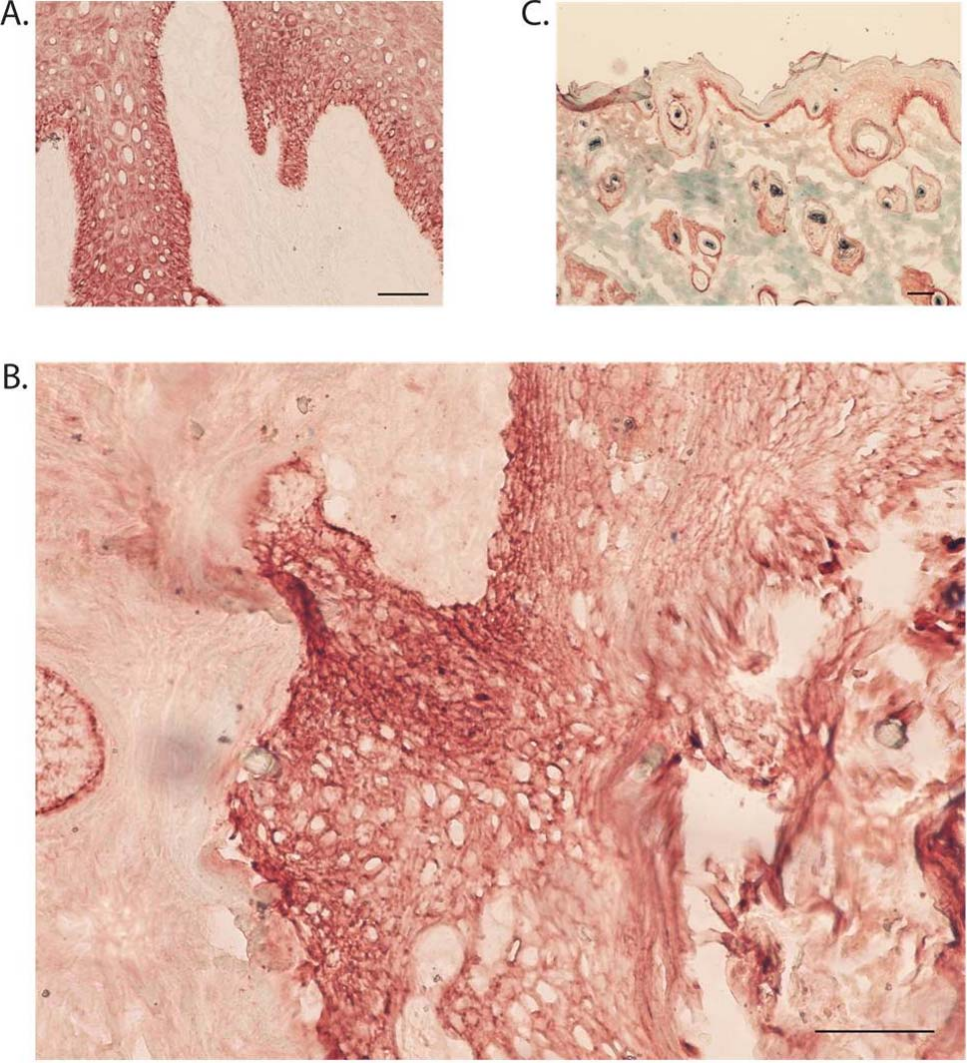
Human SCC tumor nests remain CK5/6-positive four weeks after transplantation in the nude rat. Fresh human SCC stains heavily with cytokeratin 5/6 (4A). After four weeks, tumor nests also stain heavily for cytokeratin 5/6 (4B).). Rat tissue stains in the basal layer of the epidermis only (4C).

The graft site was dissected en bloc and included rat epidermis, dermis, and subcutis, along with the original xenograft. To determine if the transplanted tissue retained characteristics of human SCC, histological sections were examined for markers of both human antigens and SCC. Transplanted tissues were compared to an additional section of each tumor which had not been transplanted, and is designated as “fresh”. Sections stained with hematoxylin and eosin (H&E) demonstrated a clear demarcation between human and rat tissue, as indicated by architectural differences (Figure 2a). The nodule was a heterogeneous mass of fat, fibrous tissue, and tumor nests, with no evidence of necrosis (Figure 2b). Tumor nests could be identified based on histological observation of groups of atypical squamous cells with some retained differentiation including keratin pearls. All tumor nests stained positive for Human Leukocyte Antigen (HLA) Class I, which confirmed human origin. Figure 3 shows staining on fresh tumor (a), tumor after extraction (b), and rat tissue (c). As expected, rat tissue did not stain with HLA Class I. Figure 4 shows staining for cytokeratin (CK) 5/6, confirming SCC. All tumor nests from both fresh and grafted SCC stained diffusely positive for CK5/6. Rat tissue shows some nonspecific CK5/6 staining in the basal layer, (Figure 4C). These results confirm that human SCC was present at 4 weeks.

### Immunohistochemical analysis for PCNA, TUNEL, and VWF suggests tumor expansion

**Figure 5:**
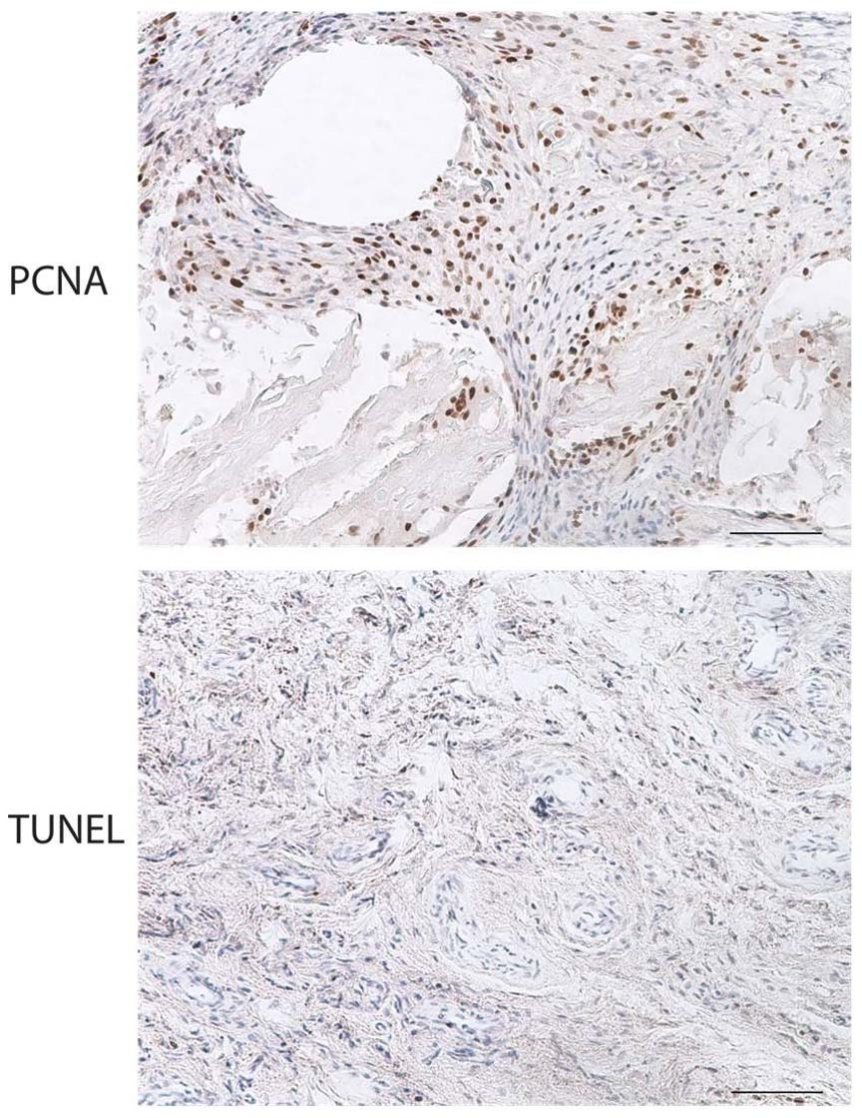
Both proliferation and apoptosis are found in transplanted SCC. PCNA was used to demonstrate proliferation and the TUNEL assay was used to visualize apoptosis. PCNA staining demonstrated active proliferation in the transplanted SCC tissue (5A). TUNEL staining demonstrated low levels of apoptosis (5B). Scale bars  = 100 µm.

**Figure 6:**
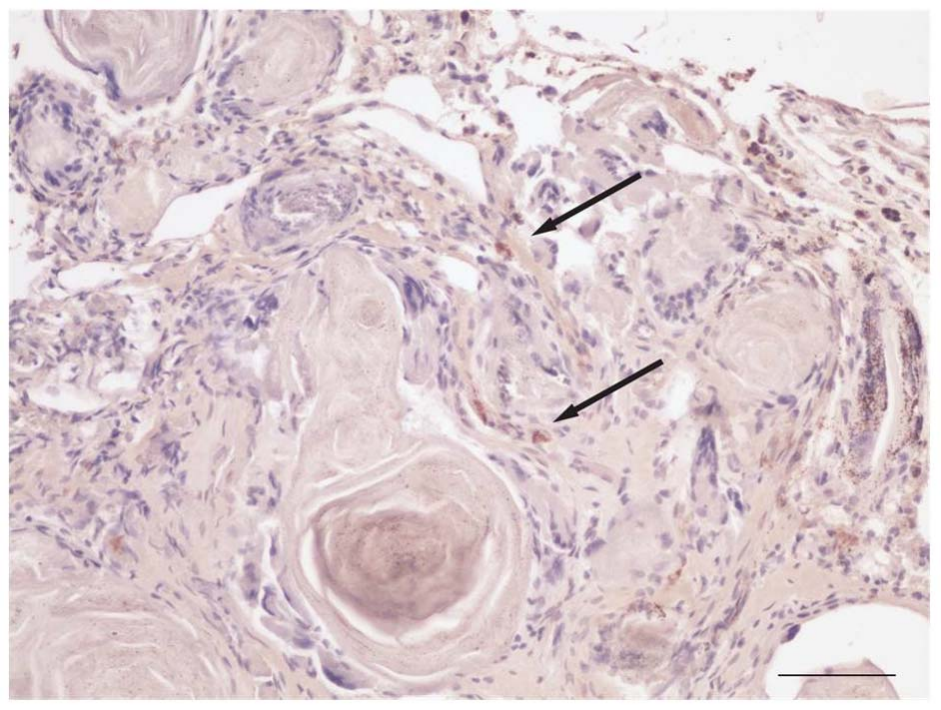
Blood vessels can be found in tumor explants after four weeks. Blood vessels were identified with staining for von Willebrand Factor and are likely from rat vessel ingrowth. Scale bars  = 100 µm.

To determine if there was active proliferation or apoptosis in the explant, immunohistochemical analysis for either proliferating cell nuclear antigen (PCNA) or TUNEL (**T**erminal deoxynucleotidyl transferase **U**TP **n**ick **e**nd **l**abeling) was performed. PCNA staining revealed proliferating cells within xenografts, corresponding to SCC on H&E (Figure 5). In high-density areas, the number of PCNA-positive cells in grafted skin was 67.6±10.3 cells/mm^2^ tissue. This value was greater than the number of PCNA-positive cells observed in fresh tumor (48.8±13.5) but was not statistically significant. TUNEL staining revealed low levels of apoptosis, especially in PCNA-dense areas (Figure 5). Numbers of TUNEL positive cells remained similar in both pre implant and post harvest tissues (21.4±8.3 vs. 24.0±5.0 cells/mm^2^ tissue). Von Willebrand's Factor (VWF) staining was used to identify vessel density in the tumor and labels both rat and human vessels. Scattered vessels were found in all harvested tumor xenografts (Figure 6). Given that PAL-E staining for human vessels was negative (data not shown), VWF positivity likely represents rat vessel in-growth.

### Human Langerhans cells remain in the tumor

**Figure 7:**
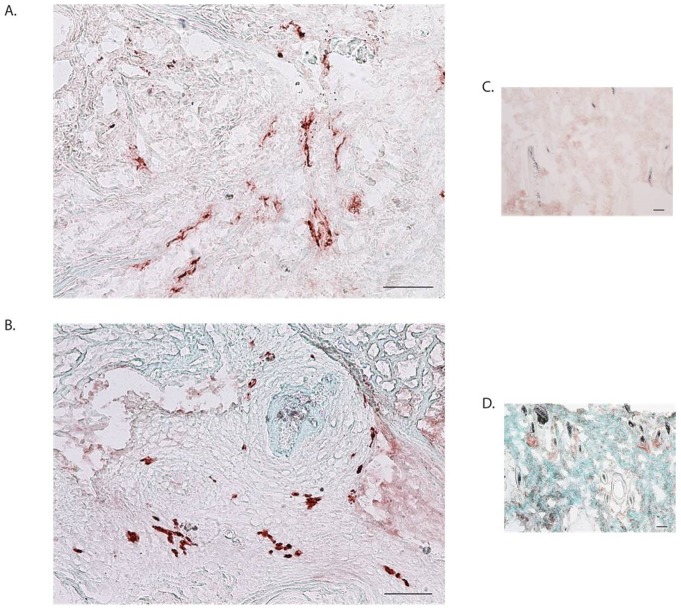
Human antigen-presenting cells survive in the tumor after four weeks. After four-DR+ cells (7A). They also contain multiple cells positive for Langerin (7B) Rat tissue does not contain any HLA-DR+ (7C) or Langerin+ (7D) cells. Scale bars  = 100 µm.

We sought to characterize the immune cell presence in tumors by immunostaining. There were numerous HLA-DR+ cells in seven of eight tumors, indicating that human antigen presenting cells (APCs) remain in the tumor (Figure 7a). There was negligible staining for dendritic cell markers CD11c and BDCA1, but seven of the eight tumors had dense staining of Langerin-positive cells, suggesting that these surviving APCs are Langerhans cells (Figure 7b). There was no staining for HLA-DR or Langerin in rat tissue (Figure 7c, d), confirming specificity for human antigens. There was negligible staining for T cell markers CD3, cytotoxic T cell marker CD8, regulatory T cell marker FoxP3, macrophage marker CD163, myeloid dendritic cell marker CD11c and resident myeloid dendritic cell marker BDCA1 (Figure S1). There was no staining for antigen presenting cell maturation marker CD83, and human and rat NK markers NKp46 and ANK61 respectively (data not shown). These results suggest immature Langerhans' cells remain in the tumor after four weeks.

## Discussion

SCC affects over 300,000 people in the United States each year and represents a significant public health burden with regard to morbidity and cost [23]. The mechanisms governing progression to metastasis are not completely understood and the treatment of lesions that have progressed beyond local surgical excision is associated with significant morbidity and mortality. While SCC is usually cured by surgical excision with clear margins, some cases behave aggressively and metastasize, despite seemingly adequate removal. Currently available therapy is inadequate for widespread or otherwise inoperable primary SCC and for metastatic SCC. Ten-year survival is only 20% with regional lymph-node involvement and less than 10% with distant organ involvement [24], and SCC makes up the majority of ∼9000 deaths per year in the US from non-melanoma skin cancer [25]. The death rate associated with aggressive cutaneous SCC parallels that of invasive melanoma [23]. Novel treatment approaches are necessary, but these require an appropriate model for testing.

Thus an SCC animal model that incorporates human tissue would provide an advantage. We have developed such a model, using tissue obtained from Mohs surgical discard. Previous animal models of SCC have not used en bloc patient-derived tumors for xenografting. Traditional models have induced skin tumors on mice via chemical carcinogenesis. These allow study of *de novo* cancer development and employ an intact tumor-host interaction. In terms of therapeutics, however, they have limited translational potential, given inherent differences between human and murine cells, immune systems, and physiology. Models using human cells tend to depend on subcutaneous inoculation of immune deficient rodents with human cancer cell lines such as HaCaT or Colo19 [3], [4], [5]. Cell lines, however, are different from native tumors. Cells from many native tumors actually do not show properties traditionally used to identify malignancy *in vitro*, such as immortalization and anchorage-independent growth [2]. In fact, metastatic SCC cells fail to grow indefinitely in culture and lack the capacity for anchorage-independent growth on soft agar [26]. Cell line models also fail to include stromal and architectural elements that may be important to cancer behavior. To address this deficiency, Khavari and colleagues [2] created a model for the study of epithelial cancers whereby regenerated human skin genetically altered for cancer growth is grafted in immune-deficient mice. This model removes variables derived from the complexity of naturally occurring tumors to allow isolation of genetic mechanisms of cancer progression. This model is very useful for studying mechanisms related to the specific genes introduced; however, it is limited as such by its focus on artificially introduced genes or mutations [2]. Our model differs by using intact naturally occurring human tumors. We see our model as more accurately recapitulating the architecture and microenvironment of naturally occurring SCC.

Our experiments demonstrate that SCC taken directly from human skin can be successfully transplanted in nude rats, with excellent viability and continued growth. Our model is based directly on previous work showing successful transplantation of full-thickness neonatal human skin to nude rats with no evidence of early rejection or necrosis [27]. The subcutaneous grafts in the present study were similarly viable. This was evident by adherence to the rat tissue bed, a tumor-like gross and histological appearance, no gross or histological evidence of necrosis, identification of pockets of proliferating cells, evidence of neovascularization, and low numbers of apoptotic cells. Furthermore, immune staining for HLA I and CK5/6 identified the persistence of human squamous cell carcinoma.

Animal models have had more extensive study and use in SCC of the head and neck (HNSCC), specifically of the upper aerodigestive tract. Models similar to ours show persistence of solid tumor after subcutaneous grafting, with retention of histological characteristics similar to human HNSCC [1]. Others use orthotopic rather than subcutaneous grafting, with the advantage of more closely simulating naturally occurring epithelial cancer, but with the disadvantage of a more difficult procedure [1]. Our group found that grafting in a subcutaneous position is highly preferable as it protects the graft from cannibalization by the host (27). There have also been HNSCC models using SCID rather than nude rodents, which allow for more aggressive tumor growth but which require more difficult maintenance.

All animal models have limitations with regard to simulating human cancer. While our model incorporates stromal architecture and elements to a far greater extent than cell line models, architecturally it remains only an approximation of naturally occurring human SCC. Firstly, the subcutaneous space is an artificial microenvironment where cutaneous SCC does not develop spontaneously [2]. This is a common choice for animal models nevertheless, because of its accessibility and convenience. Secondly, though our explants retain their epithelial basement membrane zone, *it theoretically* has a less important role once transplanted to the subcutaneous space. This is an issue insofar as basement membrane integrity contributes to malignancy, which is the case in a wide range of epithelial cancers such as breast, prostate, lung, kidney, and skin [28]. Since in this case we select tumors based on invasive histology, however, the basement membrane is compromised from the start and we need not rely on its integrity to modulate malignancy.

We noted volume contraction after 28 days however human SCC cells continued to proliferate and apoptosis remained at pre implantation levels. Volume contraction may have been to an initial lag in engraftment. We will address this and other issues as we develop the model further and extend the time course.

Another limitation to this model is the absence of metastasis. This preliminary study was only four weeks, which is a short timeframe to show metastasis. It is likely necessary to extend the time course to fully realize the potential of the model. It may be challenging to achieve metastasis with implantation based models. For example, Ras-driven human SCC lethally disseminates when cells are injected into the bloodstream but not when they are implanted above a regenerated basement membrane [29], [30]. This is a sure limitation for models that seek to recreate aggressive SCC from *de novo* carcinogenesis and from cell lines. However, since tumors in our model are selected based on aggressive characteristics at baseline, we can be confident at least that what survives in the rat represents human SCC.

We have previously shown that human SCC explants contain populations of myeloid dendritic cells [18], macrophages [17], Langerhans' cells [18], [31] and effector T cells including Th1, Th2, Th17, Th22 and Tc22 [31]. Tumor nests in the present model survive largely without resident inflammatory cells. Some HLA-DR-positive cells survive 4 weeks after implantation, suggesting that human antigen presenting cells remain in the tumor. Cells in the tumor also stain positive for CD207, a marker for Langerhans cells (LCs), the antigen-presenting cells of the epidermis. Murine LCs show a half life of 58–72 days [32]. Human LCs require cognate interaction with CD4+ T cells to escape apoptosis before 16 days [33]. It is possible that the LCs observed in our system interacted with CD4+ T cells prior to explantation into the nude rat. We previously found that LCs are present in SCC nests [18]. We also found previously that SCC is associated with increased lymphatic vessel density. Our present data showing that LCs remain in SCC explants after 1 month might indicate a failure of migration, a failure of lymphatic vessel connection or both. We had previously shown that LCs from SCC are potent initiators of type 1 responses which might be beneficial to antitumor immunity [31]; despite this, SCC still occurs. Our findings may provide some preliminary evidence that the failure of Langerhans cells to stimulate an anti-tumor response in SCC, is not due a functional deficit, but rather that they are not migrating out of tumors to local nodes where they could present tumor antigen. The other significance of LC survival is that if human immune cells are able to survive within the subcutaneous space of the nude rat, this would be important for adoptive immunotherapy testing that will require their reintroduction into this space.

In conclusion, this study describes the successful transplantation of patient-derived whole human SCC tumor into nude rats. We hereby establish a model in which grafts may be maintained for extended evaluation in a living system in order to study potential therapeutics in a controlled, reproducible manner.

## Materials and Methods

Institutional review board approval (Weill Cornell Medical College; Rockefeller University, NYU-Langone Medical Center) and informed consent was obtained before enrolling patients in this study, and the study adhered strictly to the Declaration of Helsinki Principles.

### Animals

All animals were maintained in compliance with the Guide for the Care and Use of Laboratory Animals prepared by the National Institutes of Health (National Institutes of Health publication no. 80–23, revised 1985). The Animal Care and Use Committee of the Weill Medical College of Cornell University approved all procedures. Congenitally athymic, homozygous, outbred, nude rats (strain NIHRNU-M) were obtained from Taconic (Germantown, NY). All nude rats weighed between 150 and 300 g and were 6 to 12 weeks of age. The nude rats were anesthetized with intraperitoneally administered ketamine (90 mg/kg body weight) and xylazine (4 mg/kg body weight) for all surgical procedures. Povidone-iodine solution was used to prepare the surgical site.

### Human Tissue Preparation

Intact specimens of SCC (n = 10) were obtained from sun-exposed skin of de-identified immune competent patients who were undergoing Mohs micrographic surgery. All histology samples in the study were reviewed by a board-certified dermatologist and ACMS certified Mohs surgeon (Carucci). Samples were selected based on invasive histology. The tumor was initially prepared by removing any necrotic tissue from the surface. Before implantation, small pieces of the tissue were removed to prepare for frozen and paraffin preservation.

### Engraftment

Ten tumors were implanted subcutaneously in ten nude rats. We followed our group's protocol for human full-thickness skin transplantation as described previously [27]. Transplantation was achieved by placing whole human SCC tumors in a subcutaneous position on the dorsal thorax of the nude rats. The subcutaneous graft bed was prepared through a 3-cm midline incision made on the dorsal thorax of each rat. The subcutaneous tissues were dissected down to the level of the panniculus carnosus with sharp and blunt dissection. The human specimen was placed on top of the panniculus carnosus with the graft dermis in apposition to the rat fascia and secured without tension with interrupted 5–0 chromic sutures (United States Surgical Corp.). The rat skin was closed with skin clips and/or suture. The incision was covered with bacitracin ointment and an occlusive dressing. As explained in Petratos *et al.*, [27] human foreskin grafted in an external cutaneous position was found to be uniformly nonviable due to the lack of an appropriate occlusive dressing and cannibalization by the host.

### Extraction

After 28 days, mice were euthanized by pentobarbital injection. The dorsal incision was opened with a scalpel. Human tissue survival was grossly assessed with respect to tissue morphological features, color, and adherence to the graft bed. Graft tissue was removed from the wound site en bloc. Dimensions (width × length × thickness) were measured and compared to dimensions pre-engraftment. Half of the specimen was fixed and embedded in paraffin and half was prepared for frozen preservation. Hematoxylin and eosin (H&E) staining and immunohistochemical analyses were performed to confirm the presence of SCC and assess cellular proliferation, vascular presence, and remaining inflammatory infiltrate within the grafts.

### Immunohistochemical analysis in frozen sections

Standard procedures were used for immunohistochemistry as previously described [18]. Sections of fresh tumor (n = 8) and explanted tumor (n = 8) were stained for HLA I (n = 8), CK5/6 (n = 8), HLA-DR (n = 2), CD207, CD11c, BDCA1, CD163, CD83, CD3, CD8, FoxP3, NKp46, and ANK61 (antibody information can be found in Table S1).

### Immunohistochemical analysis in paraffin sections

Proliferating cell nuclear antigen (PCNA): staining for PCNA was performed as previously described by our group [34]. Embedded tissue was deparaffinized with CitriSolv (Fisher Scientific, Fair Lawn, NJ) and ethanol. Next, antigen retrieval was performed with 0.1 M citrate buffer (pH 6.8). Endogenous peroxidase was quenched with 0.3% H_2_O_2_ in methanol. Slides were then blocked for 30 minutes with 5% bovine serum albumin solution. Slides were incubated with PCNA-specific monoclonal antibody (Dako, Carpinteria, CA) at 1∶400 for 1 hour at room temperature. Secondary and tertiary antibodies from the Vector Kit (Vector Laboratories, Burlingame, CA) were incubated at room temperature for 30 minutes each. The samples were counterstained with hematoxylin (Fisher Scientific). The number of PCNA^+^ cells was quantified in 10 high-power fields in three different specimens each of fresh tumor and explanted tumor.

Von Willebrand Factor (vWF): samples were initially deparaffinized with Hemo-de and ethanol. Endogenous peroxidase was quenched with 0.3% H_2_O_2_ in methanol. Antigen retrieval with 0.1% trypsin (ICN Biomedicals, Aurora, OH) and 0.1% CaCl_2_ (pH 7.8) was then performed for 10 minutes. Blocking was performed with 5% bovine serum albumin for 30 minutes. Polyclonal antibody to factor VIII (vWF; Dako) was added at 1∶200 and incubated for 1 hour at room temperature. Secondary and tertiary antibodies from the Vector Kit (Vector) were applied and incubated at room temperature for 30 minutes each. Finally, the samples were counterstained, as described above.

### Statistical analysis

T- tests were used to determine significance of cell counts pre and post explant. Significance was defined as P<0.05.

## Supporting Information

Figure S1 **There is significant staining for CD3, CD8, FoxP3, CD11c, BDCA1 and CD163 in SCC prior to implantation.** At day 28 post explant, there are few to no CD3+, CD8+, FoxP3+, CD11c+, BDCA1+, and CD163+ cells remaining.
(TIFF)

Table S1 (DOC)
