# Supplementary figures and images for: An Animal Explant Model for the Study of Human Cutaneous Squamous Cell Carcinoma
Source: PLoS One. 2013 Oct 8;8(10):e76156. doi: 10.1371/journal.pone.0076156 (PMC3792940; doi:10.1371/journal.pone.0076156)

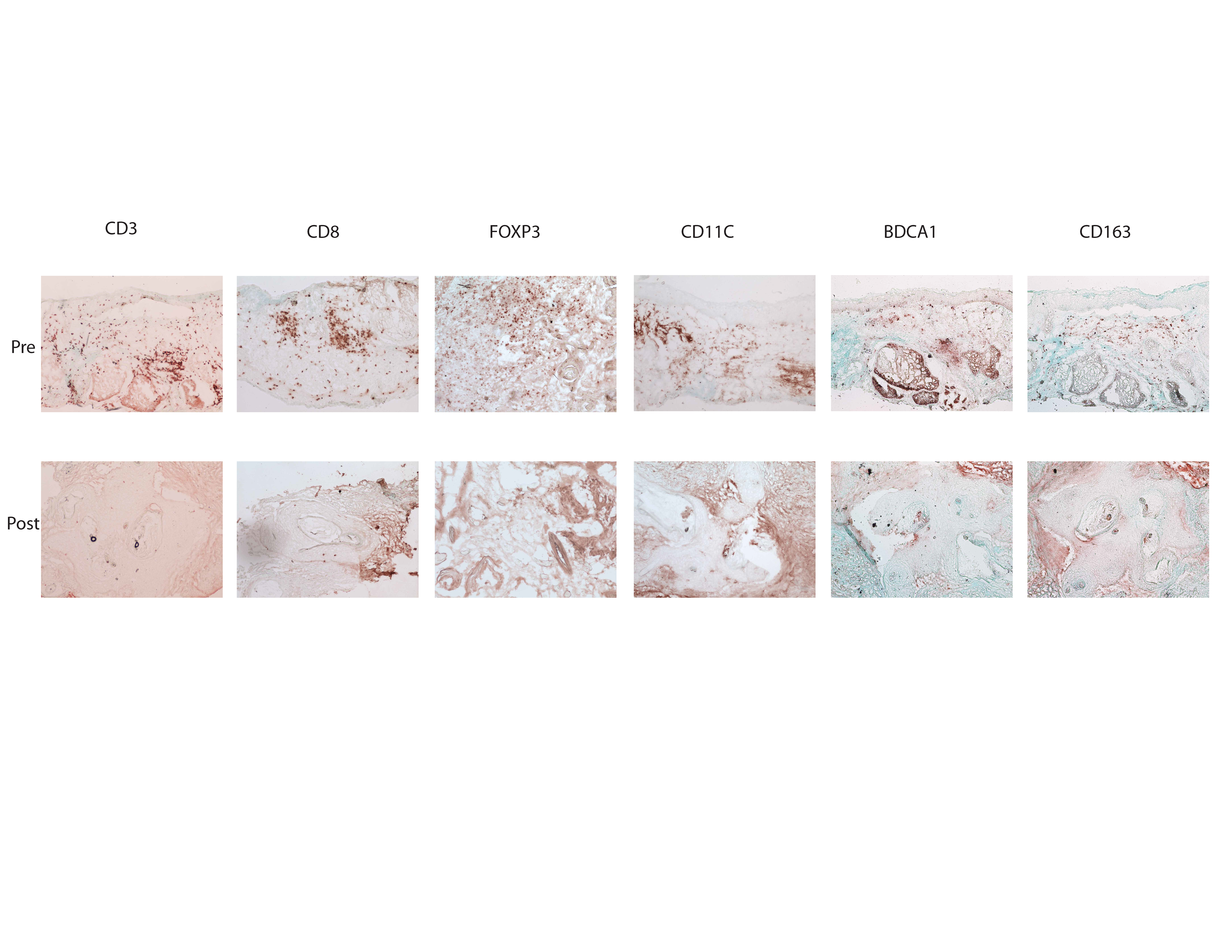

Supplement: Figure S1 — There is significant staining for CD3, CD8, FoxP3, CD11c, BDCA1 and CD163 in SCC prior to implantation. At day 28 post explant, there are few to no CD3+, CD8+, FoxP3+, CD11c+, BDCA1+, and CD163+ cells remaining. (TIFF) [file pone.0076156.s001.tiff]
