# Supplementary material for: An Animal Explant Model for the Study of Human Cutaneous Squamous Cell Carcinoma
Source: PLoS One. 2013 Oct 8;8(10):e76156. doi: 10.1371/journal.pone.0076156 (PMC3792940; doi:10.1371/journal.pone.0076156)
Supplement: Table S1 — (DOC) [file pone.0076156.s002.doc]

**Table S1: Antibodies used for Immunohistochemical analysis in frozen sections**

| **Antigen** | **Manufacturer** | **Location** | **Dilution** |
| --- | --- | --- | --- |
| CD3 | BD Pharmingen | San Diego, CA | 1:100 |
| CD8 | BD Pharmingen | San Diego, CA | 1:100 |
| FoxP3 | Abcam | Cambridge, MA | 1:40 |
| CD207 | Immunotech | Marseille, France | 1:50 |
| CD11c | BD Pharmingen | San Diego, CA | 1:100 |
| BDCA1 | Miltenyi Biotech | Auburn, CA | 1:100 |
| CD163 | Acris | Hiddenhausen, Germany | 1:100 |
| CK5/6 | Abcam | Cambridge, MA | 1:50 |
| HLA-DR | BD Pharmingen | San Diego, CA | 1:100 |
| ANK61 | Abcam | Cambridge, MA | 1:100 |
| NKP46 | R&D | Minneapolis, MN | 1:100 |
